# Population Dynamics and Nutritional Indices of *Spodoptera frugiperda* (Lepidoptera: Noctuidae) Reared on Three Crop Species

**DOI:** 10.3390/life14121642

**Published:** 2024-12-11

**Authors:** Kifle Gebreegziabiher Gebretsadik, Xiangyong Li, Yanqiong Yin, Xueqing Zhao, Fushou Chen, Hongmei Zhang, Yan Wang, Ying Liu, Gao Hu, Aidong Chen

**Affiliations:** 1 Key Laboratory of Green Prevention and Control of Agricultural Transboundary Pests of Yunnan Province, Agricultural Environment and Resource Research Institute, Yunnan Academy of Agricultural Sciences, Kunming 650205, China; 2 State Key Laboratory of Cotton Biology, Key Laboratory of Plant Stress Biology, School of Life Sciences, Henan University, Kaifeng 475001, China; 3 Tigray Agricultural Research Institute (TARI), Mekelle P.O. Box 492, Ethiopia; 4 Department of Entomology, College of Plant Protection, Nanjing Agricultural University, Nanjing 210095, China; hugao@njau.edu.cn

**Keywords:** fall armyworm, populations, life table, sorghum, sugarcane, maize

## Abstract

The fall armyworm (FAW) is an invasive pest that has been rapidly spreading across China since its detection in Yunnan province in January 2019. Although sugarcane and sorghum have been reported as hosts, their effects on FAW’s population growth and life table parameters have not been examined in China. Our research shows that FAW’s development and life table metrics vary significantly when reared on sorghum, sugarcane, and maize. Notably, the preadult stage, adult preoviposition period, and total preoviposition period of FAW were markedly longer on sugarcane and sorghum compared to maize. FAW reared on these two crops also exhibited reduced survival rates, pupal weight, fecundity, and lower female-to-male ratios. The study highlights that FAW had lower population growth rates, reproductive rates, and longer generation times on sugarcane and sorghum compared to maize. The consumption index and digestibility index were higher on maize, while conversion efficiency and growth rate were greater on sorghum. Although maize is the most favorable host, FAW can still survive and reproduce on sugarcane and sorghum during the nongrowing season, posing a risk to economically important crops in China. Despite being less favorable for population growth, sugarcane and sorghum still support FAW development and spread. Therefore, enhanced surveillance and early warning measures for sugarcane and sorghum are recommended to monitor FAW population dynamics and mitigate its potential impact on primary host plants.

## 1. Introduction

The fall armyworm (FAW), *Spodoptera frugiperda* (Lepidoptera: Noctuidae), is a prominent invasive insect pest originally from the tropical and subtropical regions of the Americas [1,2,3]. While it reproduces in these areas, it also migrates to temperate North America for summer breeding [1]. The pest was first observed in Asia in 2018 [4] and poses a significant threat to maize and other economically important plant species due to its broad host range [5,6]. FAW exhibits several invasive traits, including a lack of diapause, short generation times, high fecundity, polyphagy, and resilience against insecticides, viruses, and Bt toxins [7]. The prevalence of FAW is inconsistent, which drives long-distance migration; moths can travel over 100 km in a single night [8,9]. The number of generations FAW produces each year is influenced by environmental factors, including temperature and host plant availability [10]. For example, during the warm summer months, with daily temperatures around 28 °C, FAW has a generation time of approximately 30 to 40 days, while during cooler seasons, with daily temperatures around 17 °C, this cycle extends to about 55 days [10,11].

FAW feeds on over 353 host plant species across 76 families, including several vital crops such as maize (*Zea mays*), rice (*Oryza sativa*), wheat (*Triticum aestivum*), cotton (*Gossypium hirsutum*), sorghum (*Sorghum bicolor*), sugarcane (*Saccharum officinarum*), tobacco (*Nicotiana tabacum*), tomato (*Solanum lycopersicum*), potato (*Solanum tuberosum*), and peanut (*Arachis hypogaea*) [12,13]. There are two strains of FAW: the maize strain (sfC) and the rice strain (sfR), each exhibiting distinct preferences for host plants [14]. Whole genome sequencing and principal component analysis of the TPI gene (*triosephosphate isomerase*) suggest that the invasive populations of FAW are derived from the maize strain [14], making maize its primary host. The first report of FAW in China was recorded in January 2019 in Yunnan province [15]. This pest primarily impacted maize but also caused some damage to rice, wheat, sorghum, and sugarcane, albeit to a lesser extent [16]. Furthermore, FAW larvae have been observed on grassy weeds such as hairy crabgrass (*Digitaria sanguinalis*), Sudan grass (*Sorghum sudanense*), and goosegrass (*Eleusine indica*) [17].

Many farmers rely on synthetic insecticides to control FAW populations [18]. However, the emergence of pesticide resistance and the concealed feeding habits of FAW larvae limit the effectiveness of these insecticides [19,20,21]. Host plant resistance refers to the heritable traits that allow a plant species to minimize its vulnerability to being utilized as a host by an insect species [22,23]. This resistance is a crucial component of the integrated pest management (IPM) strategy to mitigate plant damage caused by insects like FAW [10]. It includes utilizing native genetic resistance, developing germplasm resilience to insect pests, and transferring resistance genes from other host plants to improve the recipient plant’s defenses against insect threats [23].

FAW impacts many crops and grasses in China, including maize, rice, wheat, sugarcane, and sorghum [24]. However, research on FAW population dynamics has primarily concentrated on maize and wheat, with relatively few studies focused on sorghum and sugarcane. These plants possess similar morphological characteristics and belong to the same family as maize. In Yunnan province, sorghum and sugarcane are cultivated for various purposes, such as food, animal feed, brewing, and industrial applications [25,26]. The FAW life table provides critical data on population parameters, including mortality, fecundity, age distribution, and life expectancy [27]. Host plant species’ differences significantly influence FAW’s survival rates, growth, and fecundity [28,29]. This study first examined FAW’s life table and population dynamics when fed on maize, sorghum, and sugarcane. Additionally, we assessed the feeding preferences and nutritional indices of 4th instar FAW larvae across these three plant species. This research enhances our understanding of the potential damage caused by FAW invasions. Moreover, the findings offer insights into how this pest reacts to various host plants with distinct nutritional qualities and its preferences for specific plants, which will aid in future management strategies.

Studying how FAW can utilize these different host plants offers valuable insights into their adaptive capabilities. This reveals the pest’s ability to overcome plant defenses and diversify its feeding sources—a critical aspect of understanding its biology. It helps to identify the range of host plants that FAW can utilize. Alternate hosts may serve as refuges or bridging hosts when the primary host is unavailable. Recognizing these alternate hosts helps in close monitoring and effectively managing pest populations. Furthermore, studying the interactions between FAW and their host plants can indicate the potential FAW pressure on various crops, even those not considered primary hosts. This information is crucial in anticipating and preparing for pest outbreaks, especially when the preferred host is scarce. Researchers and growers can develop more targeted and proactive management strategies by comprehensively understanding an FAW’s host plant preferences and adaptability. This knowledge is essential for avoiding potential pest challenges and mitigating their impact on agricultural systems. Thus, this study aimed to examine the effects of sorghum, sugarcane, and maize on the life table and nutritional index of FAW under controlled conditions.

## 2. Materials and Methods

### 2.1. Insects and Plant Species

The laboratory experiments were conducted at China’s Agriculture Environment and Resources Institute of the Yunnan Academy of Agricultural Sciences (YAAS). The (FAW) specimens used in this study were collected from maize fields in Songming County, Kunming City, Yunnan, in July 2022. These insects were reared on maize seedlings in the laboratory for over ten generations. They were maintained in a growth chamber set to a temperature of 25 ± 1 °C, a relative humidity of 75 ± 5%, and a photoperiod of 16 h of light followed by 8 h of darkness.

The host plant species, including sorghum, sugarcane, and maize, were used for the population growth and life table study. Seeds from all plant species were obtained from YAAS, and each species was planted separately in plastic pots (10 × 15 cm^2^) filled with a 3:1:1 mixture of organic soil, peat, and vermiculite.

### 2.2. Life Table Study of Fall Armyworm

Life table studies of the FAW fed on maize, sorghum, and sugarcane were conducted following the method outlined by Gebretsadik [30]. Three egg masses containing 334 eggs laid within six hours were collected from the climate room and placed in a Petri dish until hatching. A moistened filter paper was added to maintain elevated humidity levels (approximately 60–70% RH). The eggs were inspected every 6 h, and the number of hatched larvae was recorded. Individual 1st instar larvae were transferred from each Petri dish to a plastic cup (3 × 4 × 3.5 cm^3^) containing stems and leaves using a soft camel hairbrush. The number of 1st instar larvae transferred to maize, sorghum, and sugarcane was 76, 141, and 117, respectively. The stems and leaves provided to the larvae were replaced every 24 h to prevent fungal contamination. Larval survival and development were monitored and recorded daily. Newly pupated larvae were collected every 24 h, at which point they were sexed, weighed, and kept separately in plastic cups lined with cotton until they emerged as adults. Once the adult moths emerged, they were paired by sex and housed in transparent plastic cylindrical boxes (8.5 × 6 cm^2^). Pairs were provided with a cotton ball soaked in 10% sucrose water for nutrition and a piece of creased buffer paper as an oviposition substrate. The buffer paper and cotton ball were replaced daily until the female moth died. If the male died first, a new male from the same mass-reared colony was introduced until the female’s death. Newly laid eggs were collected and counted daily. These experiments were carried out in climatic chambers under controlled conditions: a temperature of 25 ± 1 °C, a relative humidity of 70% ± 5%, and a photoperiod of 16 h light and 8 h darkness.

### 2.3. Life Table Data Analysis

The age-stage two-sex life table data were analyzed using the TWOSEX-MSChart-Version 2024.12.01 program [31], following the methodology established by [32,33]. The population parameters evaluated included the age-stage-specific survival rate (*s_xj_*) (*x* = age, *j* = stage), age-specific survival rate (*l_x_*), age-stage-specific fecundity (*f_xj_*), age-specific fecundity (*m_x_*), age-stage life expectancy (*e_xj_*), and age-stage-specific reproductive value (*v_xj_*). Additionally, life table parameters such as gross reproductive rate (GRR), net reproductive rate (*R*_0_), intrinsic rate of increase (*r*), finite rate of increase (*λ*), and mean generation time (*T*) were also calculated [32].

The age-specific survival rate (*l_x_*) was calculated using the following formula:

(1) $$l_{x}=\sum_{j=1}^{m}s_{xj\;}$$

where *m* is the number of developmental stages.

Age-specific fecundity (*m_x_*) was computed as follows:

(2) $$m_{x}=\frac{\sum_{j=1}^{m}s_{xjf_{xj}}}{\sum_{j=1}^{m}s_{xj}}$$

where *m* is the last stage of the study cohort.

The intrinsic rate of increase (*r*) was analyzed using the Euler–Lotka equation, with age indexed from 0, as the following equation [34].

(3) $$\sum_{x=0}^{\infty }e^{-r_{m}\left(x+1\right)/m_{x}=1}$$

The net reproductive rate (*R*_0_), which represents the total number of offspring an individual can produce over its lifetime, was calculated using the following formula:

(4) $$R_{o}=\sum_{x=0}^{\infty }l_{x}m_{x}$$

The finite rate of increase (ƛ) was determined as follows:

(5) $$ƛ=e^{r_{m}}$$

The mean generation time (*T*) indicates the duration a population needs to increase to *R*_0_-fold its size as time approaches infinity, settling into a stable age-stage distribution. It was calculated as follows:

(6) $$T=\frac{lnR_{0}}{r_{m}}$$

Age-stage-specific life expectancy (*e_xy_*), representing the expected lifespan of an individual of age *x* and stage *y*, was calculated using the method described by [35]:

(7) $$e_{xy}=\sum_{i=x}^{n}\sum_{j=y}^{m}{s^{\prime }}_{ij}$$

where ${s^{\prime }}_{ij}\;$is the likelihood that an individual of age *x* and stage *y* survives to age *i* and stage *j.*

The means and standard errors for all population growth and life table parameters were analyzed using the bootstrap method, involving 100,000 repetitions (B = 100,000) [31]. The paired bootstrap test technique was employed to assess differences between treatments based on confidence intervals [36].

### 2.4. Food Consumption and Utilization

Food consumption and utilization metrics were assessed following the methodologies outlined by [37,38], utilizing the dry weights of each treatment. Newly hatched larvae (<24 h) were maintained on the stems and leaves of each host plant up to the 4th instar stage. Newly molted 4th instar larvae starved for 24 h were weighed and placed individually into plastic cups (6 cm diameter × 1.5 cm height) with a holed cover for ventilation and were supplied with fresh stems and leaves from each experimental plant. A filter paper was placed at the bottom of the Petri dish, and drops of water were added to ensure humidity. The larvae were monitored and provided with fresh stems and leaves daily. After three days, the larvae, remaining stems and leaves, and feces were separated, weighed, and dried in an oven at 60 °C for 72 h until reaching a constant weight and then weighed. Weight gain was determined by calculating the difference between the final larval and initial weights at the start of the fourth instar. The weight of eaten food was calculated as the difference between the weight of newly served food and the leftovers found the next day.

The food utilization indices, including consumption index (CI) = E/A, approximate digestibility (AD) = E − F/E, efficiency of conversion of ingested food (ECI) = P/E, efficiency of conversion of digested food (ECD) = P/E – F, relative consumption rate (RCR) = E/A × T, and relative growth rate (RGR) = P/A × T, of FAW were calculated according to Waldbauer and Han [37,39] on maize, sorghum, and sugarcane stems and leaves.

Where,

A = dry weight of the insect over unit time;

E = dry weight of food consumed;

F = dry weight of feces produced;

T = duration of feeding period; and

P = insect dry weight gain.

### 2.5. Statistical Analysis

The means and standard errors of FAW pupal weight reared on the different host plant species were subjected to one-way analysis of variance (ANOVA), and an independent samples *t*-test was used for the male and female pupae from the same plant species data to analyze the significance at *p* < 0.05 using SAS version 9.2 software [40]. All graphs were produced using the GraphPad Prism 8.0 tool [41].

## 3. Results

### 3.1. Developmental Duration and Adult Longevity of Fall Armyworm

The developmental period and adult longevity of FAW were evaluated on sorghum, sugarcane, and maize, as shown in Table 1. The findings indicate no significant differences in the egg-hatching period among the host plant species tested. However, these host plant species significantly affected other FAW developmental and longevity parameters (*p* < 0.001). Notably, the larval periods on sugarcane (29.39 d) and sorghum (28.35 d) were significantly greater than that on maize (13.72 d). The duration of the 1st, 2nd, 3rd, 4th, 5th, and 6th larval instars were substantially greater on sugarcane and sorghum than on maize. Furthermore, the 4th, 5th, and 6th larval instars were significantly prolonged on sugarcane relative to sorghum. The pre-pupal stage, total pre-adult period, adult lifespan, and overall longevity of FAW were also significantly longer for individuals who fed on sugarcane and sorghum than those fed on maize (*p* < 0.05).

### 3.2. Pupal Weight

**Figure 1:**
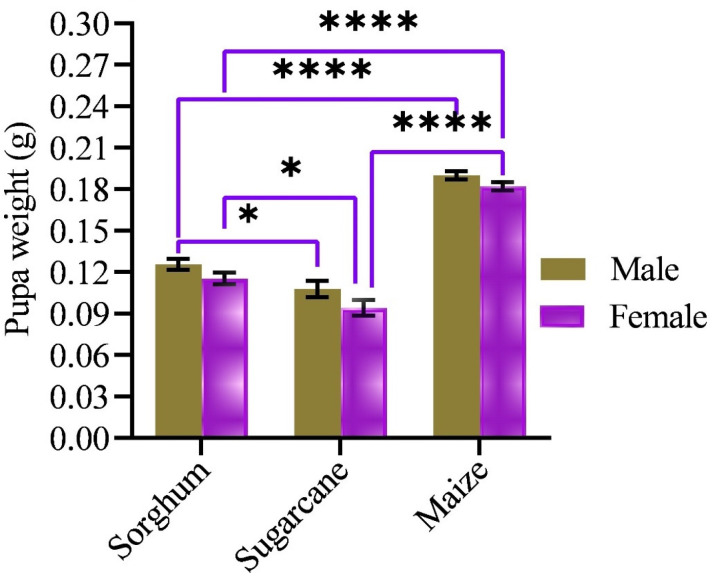
Pupa weight (M ± SE) of FAW fed on sorghum (male, *n* = 38; female, *n* = 36), sugarcane (male, *n* = 15; female, *n* = 10), and maize (male, *n* = 33; female, *n* = 32). The asterisk indicates the significant difference (*, *p* < 0.05; ****, *p* < 0.0001).

The study revealed that the pupal weight of FAW varied significantly among specimens reared on the three plant species, as shown in Figure 1. The pupal weight was higher on maize than on sorghum and sugarcane (*p* < 0.001). However, no statistically significant difference in pupal weight was observed between male and female FAW within the same host plant species. These results suggest that the choice of host plant species affects FAW pupae’s biomass accumulation.

### 3.3. Reproduction Parameters of Fall Armyworm

The results summarized in Table 2 revealed that the host plants significantly influenced the reproductive capacity of FAW. Individuals fed on sugarcane and sorghum exhibited a considerably longer average duration of pre-oviposition (APOP) and total pre-oviposition (TPOP) compared to those fed on maize (*p* < 0.001). Additionally, the oviposition period was relatively shorter for FAW on sugarcane (5.20 days) when compared to sorghum (6.38 days) and maize (6.32 days) (*p* < 0.001). FAW exhibited significantly greater fecundity when reared on maize (1705.45 eggs) compared to those on sorghum (769.94 eggs) or sugarcane (658.00 eggs) (*p* < 0.001). The female ratio of FAW was also higher on maize (48.44%) compared to those on sugarcane (40%) or sorghum (41%). These findings suggest that the reproductive capacity of FAW is significantly diminished when reared on sugarcane or sorghum, potentially due to the inadequate nutritional content of these host plants.

### 3.4. Population Parameters of Fall Armyworm

Table 3 presents the population parameters of FAW fed on stems and leaves from maize, sorghum, and sugarcane plant species. Among the plants tested, maize showed the highest *r* (0.205 day^−1^), *λ* (1.228 day^−1^), and *T* (31.87 days). The FAW individuals fed maize stems and leaves also exhibited the highest gross reproductive rate (GRR = 906.96) and *R*_0_ (906.96) compared to those fed sorghum and sugarcane (*p* < 0.001). Conversely, FAW fed on sugarcane stems and leaves recorded the lowest values for *r* (0.075 day^−1^), *λ* (1.078 day^−1^), *R*_0_ (56), and GRR (341.95), while also exhibiting the longest *T* (53.46 days) (*p* < 0.001). These findings indicate that sugarcane is the least suitable host plant for the growth of the FAW population.

### 3.5. Survival Rate and Fecundity of Fall Armyworm

Table 4 displays the survival rates of FAW at each larval, pre-pupal, pupal, and adult stage when reared on the three host plants. Survival rates were consistently highest on maize and lowest on sugarcane across all developmental stages. When FAW larvae were fed on maize, survival rates were 86.84% for larval, 85.53% for pre-pupal, 85.53% for pupal, and 83.91% for adult stages. Conversely, the survival rates for those fed on sugarcane were significantly lower, with larval, pre-pupal, pupal, and adult survival rates at 67.52%, 47.86%, 23.93%, and 8.55%, respectively. These results suggest that FAW survival is adversely affected when reared on sugarcane compared to maize.

**Figure 2:**
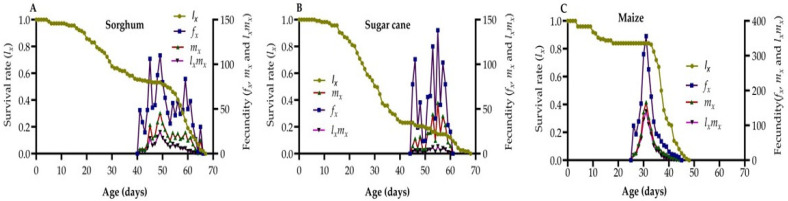
Age-specific survival rate (*l_x_*) and female age-stage-specific fecundities (*f_x_*), fecundity (*m_x_*), and net maternity (*l_x_* × *m_x_*) of FAW fed on sorghum (**A**), sugarcane (**B**), and maize (**C**).

The age-stage survival rates (*l_x_*), fecundity (*m_x_*), and net maternity (*l_x_* × *m_x_*) curves of FAW reared on three host plant species are illustrated in Figure 2A–C. The lx values declined sharply with the increasing age of all three host plants. The adult lifespan of FAW on sorghum, sugarcane, and maize was 66 days, 67 days, and 47 days, respectively. The highest peaks in *f_x_*, *m_x_*, and *l_x_* × *m_x_* were observed at 31 d on maize, with values of 356.20, 166.98, and 140.61, respectively. In contrast, the lowest peaks were recorded at 55 days on sorghum, yielding values of 110.03, 45.48, and 24.19, respectively. The *m_x_* curve on maize exhibited a single peak, indicating a distinct period of high fecundity. In contrast, sorghum and sugarcane showed multiple peaks in their *m_x_* curves, suggesting variability in adult emergence and oviposition periods among FAW individuals feeding on the same host plant species.

### 3.6. Age-Stage-Specific Survival Rate

**Figure 3:**
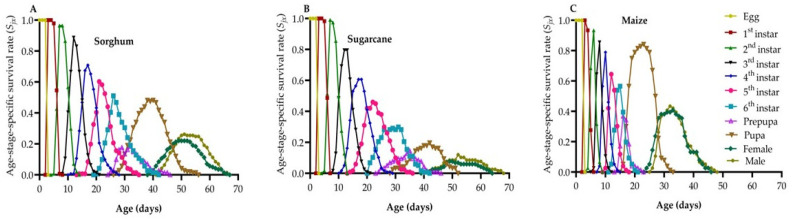
The age-stage-specific survival rate of FAW on sorghum (**A**), sugarcane (**B**), and maize (**C**). *S_xj_*: the probability that a newly laid egg will survive to age *x* and stage *j*.

The effects of host plants on the survival of newly hatched FAW at specific age (*x*) and stage (*j*) are shown in Figure 3. There was a significant overlap in survival rates due to variations in developmental rates among FAW individuals. The survival rate during the larval stage was lowest on sugarcane (29.9%) and highest on maize (60%). From the egg to the pupal stage, the survival rate was the weakest on sugarcane (23.93%) and highest on maize (85.53%). The survival rate to adulthood followed a similar pattern, with the lowest being on sugarcane (21.37%) and the highest on maize (84.21%). Additionally, male adult FAW showed longer overall survival durations than female adults on sugarcane and maize, with female adults emerging 1–2 days earlier than their male counterparts, as illustrated in Figure 3A–C.

### 3.7. Life Expectancy

**Figure 4:**
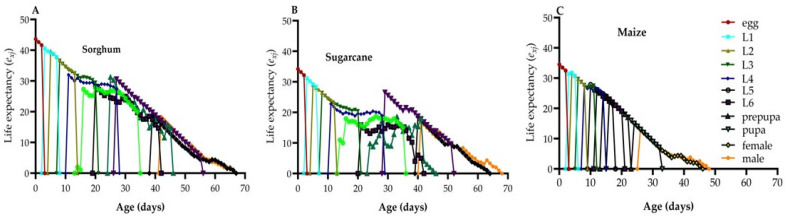
Life expectancy (*e_xj_*) of FAW on sorghum (**A**), sugarcane (**B**), and maize (**C**).

The *e_xj_* of FAW individuals is indicative of their life expectancy after reaching a specific age *(x*) and stage (*j*) (Figure 4A–C). FAW specimens fed on sorghum had the longest *e_xj_* for newly laid eggs (43.66 d) compared to those on maize (34.51 d) and sugarcane (34.20 d). The highest *e_xj_* was observed on sorghum in male adults (18.29 d) and female adults (18.96 d), followed by sugarcane males (16.97 d) and females (18.59 d). The lowest *e_xj_* were noted for males (13.00 d) and females (15.13 d) on maize. Figure 4 provides a detailed breakdown of these life expectancies for FAW specimens based on their diets, illustrating how the anticipated survival time varies depending on the host plant.

### 3.8. Reproductive Value

**Figure 5:**
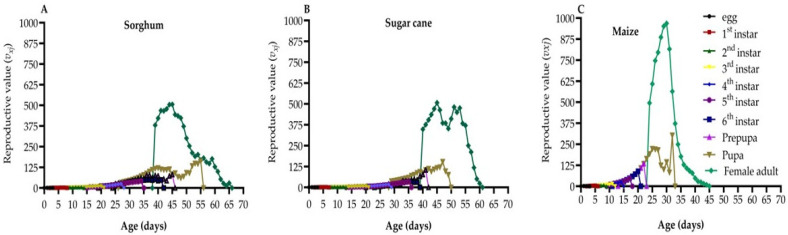
Age-stage-specific reproductive value (*v_xj_*) of FAW on sorghum (**A**), sugarcane (**B**), and maize (**C**).

The age-stage-specific reproductive values (*v_xj_*) of FAW represent the contribution of individuals at age (*x*) and stage (*j*) to the next generation (Figure 5A–C). The *v_xj_* increased substantially as FAW commenced laying eggs, with the pupal curve of *v_xj_* on maize and sorghum showing two or more peaks. Increases in *v_xj_* were observed at 39–45, 40–45, and 24–30 days on sorghum, sugarcane, and maize, respectively. Notably, the peak reproductive value for *v_xj_* on maize occurred much earlier at 30 days, reaching 968.69 eggs. However, on sorghum and sugarcane, the peak *v_xj_* occurred later at 45 days, yielding 506.32 and 507.69 eggs, respectively. These findings indicate that FAW individuals make varying contributions to the population based on their age and stage, with significant increases in reproductive value coinciding with the onset of egg-laying.

### 3.9. Food Consumption and Utilization Analysis

**Figure 6:**
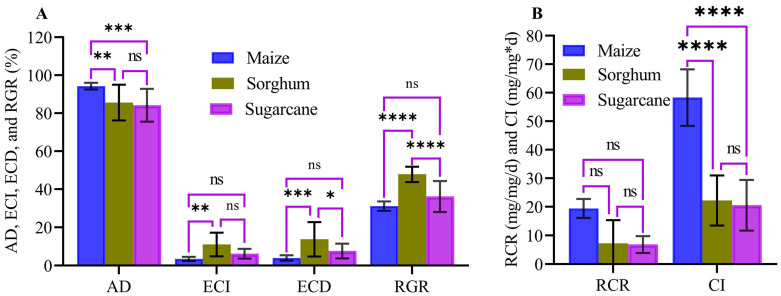
Nutrient index of fourth instar larvae and feeding preference on maize, sugarcane, and sorghum plants. Figure (**A**) illustrates approximate digestibility (AD), efficiency of conversion of ingested food (ECI), efficiency of conversion of digested food (ECD), and relative growth rate (RGR), while Figure (**B**) depicts relative consumption rate (RCR) and consumption index (CI) of FAW fed on maize, sorghum, and sugarcane.The asterisk indicates the significant difference (*, *p* < 0.05; **, *p* < 0.01, ***, *p* < 0.001, ****, *p* < 0.0001; ns, non-significant).

Results of nutritional indices for the 4th instar larvae of FAW are presented in (Figure 6A,B). The AD and CI were significantly higher in the 4th instar larvae fed on maize compared to those fed on sorghum and sugarcane (*p* < 0.05) (Figure 6A). Conversely, the ECI, ECD, and RGR were significantly higher on sorghum than on maize and sugarcane (*p* < 0.05) (Figure 6A). Although there were no significant differences in RCR among the treatments, relatively higher RCR was recorded on maize compared to sorghum and sugarcane. These results indicate that maize is more palatable to FAW larvae than sorghum and sugarcane (Figure 6B).

## 4. Discussion

Research has demonstrated that differences in host plant species significantly influence the life history of FAW and other insect pests [30,42]. As an invasive phytophagous pest, FAW can feed on more than 350 host plant species [12,43]. Numerous studies suggest that variations among the host plant species substantially impact the life history of FAW [30,42,44]. Host plant species can affect various biological parameters of insects, including growth and development rate, survival rate, and fecundity [42,45,46]. Our research findings indicate that maize is a more suitable host plant species than sorghum and sugarcane. Feeding on sugarcane and sorghum resulted in longer developmental time, reduced pupal weights, and decreased fecundity for FAW individuals, suggesting that these host plant species suppress FAW growth and reproduction. Notably, differences in the characteristics of FAW host plant species have been shown to affect the development of female reproductive organs and overall fecundity [47,48]. For instance, female FAW individuals fed on goosegrass exhibited shorter ovarioles and laid fewer eggs than those reared on maize stems and leaves [49]. These observations further highlight the significant impact of host plant species on the growth, development, and reproductive capacity of FAW and other insect pests.

FAW larvae’s nutritional status and fitness have been shown to influence various pupation parameters, including pupation rate, pupal duration, weight, and size [50]. Our research found that FAW exhibited higher population rates, survival rates, biomass accumulation, and faster development when fed on maize than sugarcane or sorghum. Furthermore, female pupae emerged as adults 1–2 days earlier than male pupae, consistent with similar findings for FAW fed on wheat and faba beans (*Vicia faba*) [30,51].

Numerous studies have reported that variations in host plant species can affect the pupal weight of FAW [30,44,49]. Insects that consume host plants with low nutritional value may experience reduced size, poor egg development, and decreased fertility [52]. For instance, Liu et al. [51] found that FAW larvae fed on maize, wheat, and alfalfa (*Medicago sativa*) had significantly heavier pupal weights than those fed on peas (*Pisum sativum*). Specifically, FAW larvae fed on sugarcane and sorghum displayed lower pupal weights than those fed on maize. However, no significant differences in pupal weight were found between male and female FAW individuals within the same host plant species, consistent with the findings reported by [44,49]. These results indicate that the nutritional composition of various host plant species can influence FAW’s developmental parameters and pupal weight, ultimately influencing their fitness and reproductive success.

The study found that FAW’s average APOP and TPOP were significantly longer when feeding on sugarcane and sorghum than maize. Similar results were reported by [31], who observed that FAW fed on maize had a longer APOP and TPOP than those fed on faba beans or soybeans, which are unsuitable host species for FAW. Thus, longer APOP and TPOP were observed on less suitable host plant species for FAW. The fecundity of adult FAW is influenced by the nutrients accumulated during the developmental stages and the nutrition provided during the adult stage [53,54]. The extended developmental period, lower pupal weight, and reduced fecundity may be attributed to differences in the carbon-to-nitrogen (C/N) ratio and metabolites utilized during larval development [55].

The population parameters, such as GRR, *R*_0_, *r*, *λ*, and *T* changes in an insect’s growth, development, reproduction, and survival, can indicate whether a population is increasing in a given environment [42,55,56]. Lower population growth rates, including GRR, *R*_0_, *r*, *λ*, and longer *T*, were observed when FAW fed on pea and faba beans compared to maize, wheat, and alfalfa [51]. Similarly, low growth rates were found for sugarcane and sorghum. In our study, the lowest values of GRR (341.95) and *R*_0_ (56) were recorded when FAW was fed on sugarcane due to reduced fecundity. This suggests that sugarcane negatively influences FAW’s reproductive capability. The findings indicated that sugarcane and sorghum are less suitable host plant species for FAW’s survival, growth, development, and fecundity.

The age-stage survival rate (*s_xj_*) curves of FAW individuals showed overlapping trends, indicating variations in developmental rates among individuals. Sugarcane had the lowest survival rates for the larval stage (29.9%), pupal stage (23.93%), and adult stage (21.37%) compared to the other host plant species tested. The *e_xj_* values of FAW exhibited a decreasing trend on all three host plant species, with the shortest average *e_xj_* value recorded on sugarcane (34.20) and the longest on sorghum (43.66). The *v_xj_* peaks occurred at 45 days, with the highest number of eggs laid being 506.32 from sorghum and 507.69 from sugarcane. The *e_xj_* is calculated based on the *s_xj_*, assuming the population maintains a constant age-stage distribution. Therefore, it may help forecast the population’s survival under those conditions.

The nutritional quality of host plants varies across different plant species for insect herbivores [57]. The dietary indices of these herbivorous insects reflect their adaptability and ability to exploit their host plants effectively [57,58,59]. In our study, the FAW’s CI, AD, and RCR were higher on maize than sorghum and sugarcane, indicating a better larval adaption. This aligns with the previously reported FAW larval preference for maize [58]. Conversely, the ECI, ECD, and RGR were greater on sorghum than on the maize and sugarcane. This suggests the FAW effectively digested and converted sorghum stems and leaves into body growth and development. Studies have shown that ECI, ECD, and RGR indicate an insect’s ability to utilize consumed food for their growth and development [58,60]. Although CI, AD, and RCR were lower on sugarcane and sorghum, the greater ECI, ECD, and RGR on sorghum indicate a more efficient conversion of ingested food into body biomass. Thus, while maize proves to be a suitable host for FAW development, sorghum and sugarcane may also function as viable alternative hosts during periods of food scarcity. Similar research has shown that the ECI and ECD of FAW on maize were lower than those on ginger (*Zingiber officinale*), suggesting ginger is also a suitable alternative host [61]. Overall, these findings underscore the potential for FAW larvae to cause significant damage to these host plants

## 5. Conclusions

The differences in host plant species significantly impact the FAW’s population growth and life table parameters. Larvae fed on sugarcane and sorghum exhibited longer durations for the larval, pre-pupal, pupal, total pre-adult, APOP, TPOP, and adult stages than those fed on maize. Furthermore, FAW individuals who consumed sugarcane and sorghum demonstrated lower survival rates, pupal weights, fecundity, CI, AD, and RCR than their counterparts fed on maize. The life table parameters for FAW fed on sugarcane and sorghum indicated reductions in GRR, *R*_0_, *r*, and *λ* and an extended *T* relative to those fed on maize. Overall, these findings underscore maize as a highly suitable host plant for the FAW, while sugarcane is the least appropriate, with sorghum positioned as intermediate. Therefore, sugarcane and sorghum provide nutritional support for FAW populations’ establishment, growth, and continued dispersal. Since sugarcane is cultivated year-round in Yunnan province, it may facilitate the maintenance and proliferation of FAW, particularly during the maize off-season. Moreover, our results indicate that FAW could inflict significant economic damage on sugarcane, sorghum, and maize. Therefore, agricultural management authorities should enhance their focus on the potential threats posed by FAW to these vital crop species.

To minimize FAW’s impact on maize, sugarcane, and sorghum crops, growers must consider rotating their cropping patterns. Instead of growing these crops in close succession or as monocultures, growers could diversify their planting schedule. For example, they should avoid planting maize immediately after sugarcane or sorghum. This can help disrupt the pest life cycle and reduce the carryover of the FAW population from one crop to the next. Incorporating other non-host crops, such as soybean, sweet potato, faba bean, and field pea, into crop rotation can enhance biodiversity and create less favorable conditions for the FAW to thrive. Adopting strategic crop rotation allows growers to manage this invasive pest effectively and safeguard yields across these important cereal and cash crops.

## Figures and Tables

**Table 1:** Developmental durations (M ± SE) of FAW on maize, sorghum, and sugarcane stems and leaves.

Duration, Days	Host Plants						
	*n*	Maize	*N*	Sorghum	*n*	Sugarcane	*p*-Value
Egg	76	3.00 ± 0.00 a	141	3.00 ± 0.00 a	117	3.00 ± 0.00 a	NS
1st instar	73	2.28 ± 0.51 b	137	3.56 ± 0.04 a	117	3.47 ± 0.05 a	***
2nd instar	73	2.11 ± 0.04 b	137	4.31 ± 0.07 a	115	4.30 ± 0.09 a	***
3rd instar	72	2.15 ± 0.05 b	134	4.70 ± 0.06 a	111	4.63 ± 0.10 a	***
4th instar	67	2.25 ± 0.05 c	128	4.79 ± 0.08 b	101	5.15 ± 0.10 a	***
5th instar	66	2.41 ± 0.07 c	112	5.01 ± 0.08 b	79	5.69 ± 0.11 a	***
6th instar	65	2.54 ± 0.07 c	93	5.53 ± 0.09 b	56	5.86 ± 0.014 a	***
1st to 6th instar	65	13.72 ± 0.18 b	93	28.35 ± 0.40 a	56	29.39 ± 0.57 a	***
Pre-pupa	65	1.40 ± 0.06 b	80	2.63 ± 0.08 a	28	2.57 ± 0.13 a	***
Pupa	64	9.69 ± 0.11 c	75	12.01 ± 0.14 a	25	10.64 ± 0.26 b	***
Total preadult	64	33.45 ± 0.55 b	75	45.88 ± 0.42 a	25	46.80 ± 0.71 a	***
All Adult	64	11.05 ± 0.35 b	75	13.43 ± 0.29 a	25	12.56 ± 0.70 a	***
Total longevity	76	39.06 ± 0.44 b	141	43.66 ± 1.53 a	117	34.20 ± 1.41 c	***

Mean values M ± SE in the same column followed by different letters were significantly different (*p* < 0.05) (paired bootstrap test). The asterisk indicates the significant difference (***, *p* < 0.001); NS, non-significant.

**Table 2:** APOP, TPOP, oviposition days fecundity (M ± SE), and female ratio of FAW when fed different host plants.

Host Plants	Biological Parameters				
	APOP	TPOP	Oviposition Days	Fecundity	Females (%)
Maize	2.84 ± 0.22 b	30.03 ± 0.33 b	6.32 ± 0.38 a	1705.45 ± 125.84 a	48.44
Sorghum	3.33 ± 0.24 a	48.39 ± 0.66 a	6.38 ± 0.38 a	769.94 ±73.11 b	41.00
Sugarcane	4.70 ± 0.65 a	50.90 ± 1.10 a	5.20 ± 0.47 ab	658.00 ± 89.67 b	40.00
*p*-value	*	***	NS	***	

Mean values M ± SE in the same column followed by different letters were significantly different (*p* < 0.05) (paired bootstrap test). The asterisk indicates the significant difference (*, *p* < 0.05; ***, *p* < 0.001). APOP, adult pre-oviposition period; TPOP, total pre-oviposition period. NS, non-significant.

**Table 3:** Population parameters (M ± SE) of FAW-fed maize, sorghum, and sugarcane.

Host Plants	Population Parameters				
	GRR	*R*_0_	*r* (Day^−1^)	λ (Day^−1^)	*T* (Days)
Maize	906.96 ± 140.99 a	695.64 ± 109.14 a	0.205 ± 0.0061 a	1.228 ± 0.007 a	31.87 ± 0.44 b
Sorghum	459.69 ± 80.71 b	196.58 ± 33.76 b	0.105 ± 0.003 b	1.110 ± 0.004 b	50.44 ± 0.87 a
Sugarcane	341.95 ± 101.91 c	56 ± 18.43 c	0.075 ± 0.007 c	1.078 ± 0.007 c	53.46 ± 1.38 a
*p*-value	***	***	***	***	***

Mean values M ± SE in the same column followed by different letters were significantly different (*p* < 0.05) (paired bootstrap test). (***, *p* < 0.001). GRR, gross reproductive rate; *R*_0_, net reproductive rate; *r* (day^−1^), intrinsic of increase; *λ* (day^−1^), finite rate of increase; *T* (days), mean generation time.

**Table 4:** Percentage survival rate of FAW at each developmental stage.

Host Plants	Survival Rate, %									
	Egg	1st Instar	2nd Instar	3rd Instar	4th Instar	5th Instar	6th Instar	Prepupa	Pupa	Adult
Sorghum	100	100	97.16	97.16	95.04	90.78	79.43	65.96	56.74	53.19
Sugarcane	100	100	100	98.29	94.87	86.32	67.52	47.86	23.93	8.55
Maize	100	100	96.05	96.05	94.74	88.16	86.84	85.53	85.53	83.91

The data in the table are the percentage survival rates of FAW at the start of each developmental stage.

## Data Availability

No new data were created.
